# Role of tissue engineered collagen based tridimensional implant on the healing response of the experimentally induced large Achilles tendon defect model in rabbits: a long term study with high clinical relevance

**DOI:** 10.1186/1423-0127-20-28

**Published:** 2013-05-14

**Authors:** Abdolhamid Meimandi-Parizi, Ahmad Oryan, Ali Moshiri

**Affiliations:** 1 Division of Surgery, Department of Clinical Sciences, School of Veterinary Medicine, Shiraz University, Shiraz, Iran; 2 Department of Pathology, School of Veterinary Medicine, Shiraz University, Shiraz, Iran

**Keywords:** Tendon, Collagen, Bioimplant, Biodegradable, Biocompatible, Healing, Rabbits

## Abstract

**Background:**

Tendon injury is one of the orthopedic conditions poses with a significant clinical challenge to both the surgeons and patients. The major limitations to manage these injuries are poor healing response and development of peritendinous adhesions in the injured area. This study investigated the effectiveness of a novel collagen implant on tendon healing in rabbits.

**Results:**

Seventy five mature White New-Zealand rabbits were divided into treated (n = 55) and control (n = 20) groups. The left Achilles tendon was completely transected and 2 cm excised. The defects of the treated animals were filled with collagen implants and repaired with sutures, but in control rabbits the defects were sutured similarly but the gap was left untreated. Changes in the injured and normal contralateral tendons were assessed weekly by measuring the diameter, temperature and bioelectrical characteristics of the injured area. Clinical examination was done and scored. Among the treated animals, small pilot groups were euthanized at 5, 10, 15, 20, 30, 40 and 60 (n = 5 at each time interval) and the remainder (n = 20) and the control animals at 120 days post injury (DPI). The lesions of all animals were examined at macroscopic and microscopic levels and the dry matter content, water delivery and water uptake characteristics of the lesions and normal contralateral tendons of both groups were analyzed at 120 DPI.

No sign of rejection was seen in the treated lesions. The collagen implant was invaded by the inflammatory cells at the inflammatory phase, followed by fibroplasia phase in which remnant of the collagen implant were still present while no inflammatory reaction could be seen in the lesions. However, the collagen implant was completely absorbed in the remodeling phase and the newly regenerated tendinous tissue filled the gap. Compared to the controls, the treated lesions showed improved tissue alignment and less peritendinous adhesion, muscle atrophy and fibrosis. They also showed significantly better clinical scoring, indices for water uptake and water absorption, and bioelectrical characteristics than the controls.

**Conclusion:**

This novel collagen implant was biodegradable, biocompatible and possibly could be considered as a substitute for auto and allografts in clinical practice in near future.

## Background

An Achilles tendon defect in the setting of large tissue loss is a more difficult problem than simple repair of a ruptured or lacerated tendon [1-3]. Due to many soft connective tissue tumors such as Xantoma, fibrosarcoma and liposarcoma, gangrenous and infective ulcers, burning, traumatic injuries (e.g. car accident or gunshot trauma), tendinitis or tendinopathies, and those neglected Achilles tendon ruptures or chronic ruptures [1,3-9], large tendon defect could occur [6]. In such circumstances, it is often necessary to resect the remaining Achilles tendon and reconstruct the defect area [6,7]. If such a treatment is neglected, then joint stiffness is developed and the functionality, especially at ankle area, would be significantly impaired which complicates the condition [4,6-8]. However, treatment of large Achilles tendon defect is technically demanding and there is no well approved gold standard method for such a large soft tissue reconstruction [2,3,6]. In fact, variation in the surgical techniques for repairing a large Achilles tendon defect is remarkable and it is therefore difficult to determine the method of choice [4,8]. Many different surgical techniques exist, only a few of them have been validated in a strict scientific manner [6]. The v-y technique, local tissue augmentation, turn-down flaps, tendon transfer, free tissue transfer and the use of synthetic materials are some of examples, each have its own significant limitations [2-4]. Tendon transplantation could be potentially a method of choice however, due to the large size of the harvesting autograft, the donor site morbidity is a major concern and the allografts have not been widely accepted as yet, due to many reasons such as disease transmission (e.g. HIV), rejection and ethical concerns [1,2]. For these reasons, treatment of such massive tendon injuries, is a state of art and depends on the surgeon’s experience, equipment, facilitation, and condition [1,3].

On the another hand, healing of the injured tendons, regardless of the surgical reconstructive methods of such large Achilles tendons defects, has some significant limitations because the tendon have low vascularity and should tolerate weight bearing forces during the healing process [5,10]. Poor healing response, development of peritendinous adhesions and low quality of the healed tendon are some examples which significantly affect the outcome of each treatment strategy [5,10].

Tissue engineering is a newer approach which aimed to assist tendon healing and make this opportunity to design newer treatment strategies [11]. By this technology, it is possible to choose a proper material and to design a newer grafts or scaffolds [11]. Several characteristics of such scaffolds could be designed based on the limitations of current treatment strategies [10,11]. An ideal scaffold which is designed for tendon tissue engineering should have similar architecture and composition as normal healthy tendon [11]. In addition, they should have optimum cell cytocompatibility *in vitro*, and have optimum biodegradability, biocompatibility and healing efficacy *in vivo*[10,11]. On the other hand, the scaffold should incorporate with the newly regenerated tissue and should not be rejected or encapsulated by the host. It should also be tenoinductive, tenoconductive and tenogenesis *in vivo*. By tenoinductivity it means the scaffold should accelerate the healing response after implantation and simultaneously chemotactically absorb the fibroblasts, preserve them and facilitate their proliferation in its architecture to induce a proper tendon healing [10]. By tenoconductivity it means such scaffold should be highly aligned to guide and organize the healing proliferating cells and the new matrix along the normal anatomical direction of the tendon which establish the tendon continuity [10]. By tenogenesis it means the scaffold should be able to be gradually replaced by the newly regenerated tissue which has similar characteristics to the normal healthy tendons [10].

In designing such a scaffold it is important to consider the architecture and composition of the normal tendons [10]. Intact tendons are mainly made up of collagen type I molecules [5]. These molecules are naturally polymerized as fibril, fiber, fiber bundles and fascicles which are arranged in a highly aligned manner [5,10]. The diameter of collagen fibrils is ranging from 30 to 300 nm while the diameter of collagen fibers and fiber bundles varies from few to several micrometers [5,10,12]. In addition, the exogenous collagen similar to the endogenous one, interacts with cells in connective tissues and transduces essential signals for the regulation of cell anchorage, migration, proliferation, differentiation, and survival [13]. Collagen is defined by high mechanical strength, good biocompatibility, low antigenicity and ability of being crosslinked, and tailored for its mechanical, degradation and water-uptake properties [14]. Collagen has been widely applied in tissue engineering applications and in some extent in delivery systems in this field [14]. Therefore, to mimic the intact healthy tendon architecture, selection of collagen molecules as a basic material for producing a new scaffold is logic. As described, the architecture of the intact tendon is a combination of nano- and micro- fibers which are highly aligned, thus it is reasonable to produce a scaffold which has a combination of highly aligned nano- and micro- fibers in its architecture.

Two different technologies are engaged in collagen tissue engineering with the aiming to reform the architecture of the scaffolds to be effective in tendon healing and regeneration. The first technology is the collagen gel system which has many advantageous [15-17]. The main advantages of gel system is its procedural simplicity and its minimal equipment requirements [15,17]. In this technology, it is possible to polymerize the collagen molecules as collagen fibers and to produce three dimensional microenvironment for cell culture and studying cell behavior [15-17]. Due to the three dimensional nature of these products they have excellent cytocompatibility *in vitro* and are biocompatible and biodegradable *in vivo*[15,16]. They can protect the cells and have proper transport properties (such as nutrients to cells or cell products from cells) [15,16].

Collagen gels has been used as dermal filler in clinical practice [15]. Their liquid form (cold neutralized collagen solutions) can be injected in the lesion; they are then polymerized as gel at body temperature [15]. Collagen gels could also be seeded *in vitro* with cells and then could be implanted *in vivo* with the aiming to repair the injured tissues [17,18]. Despite their advantages of convenience and biocompatibility, they have major limitations. For example, the inability to control final gel properties such as diameter, density and alignment of the collagen fibers is one the major limitations of this method [15]. These randomly oriented gels are too weak for surgical manipulation or in bearing tensile loads *in vivo*[19]. For tendon tissue engineering, the diameter, density and alignment of the polymerized collagen fibers should be well controlled.

Electrospinning of the collagen fibers is another approach which gives us the impression of being a very simple and easily controlled technique in producing the nanofibers [20]. By this technology it is possible to fabricate continuous collagen nanofibers with desired diameter, density and alignment to be suitable in tendon regeneration [21]. Randomly oriented electrospun fibers are produced by collecting the polymer jet on a flat sheet of grounded material [21,22]. Producing uniformly aligned fibers is accomplished by altering the collection method. Several methods have been employed to collect arrays of parallel fibers, including rotating mandrels and dual plate electrodes. Dual plate techniques has been shown to have the best impact on the alignment and density of the collagen fibers [23]. The nanofibers of the electrospun scaffolds increase the surface area of the scaffold and this increases the biocompatibility and biologic activity of the scaffold compared to those of the gel-based scaffolds [22]. However, electrospinning has some disadvantageous [21]. The major limitation of this method is the low mass production rate [20]. In tendon tissue engineering the constructed scaffold should be comparable in size with the tendon while producing a voluminous collagen scaffold by this technology increases the cost and is also time consuming [10,20,22]. Additionally, it is difficult to increase the thickness of the scaffold by this method because the electrospun scaffolds tend to be thin [20-22]. For these reasons, this technology is best suited in producing the two-dimensional scaffolds or sheets [22,23]. The major application of this technology is production of the circular tube shaped scaffolds in cardiovascular surgery. Such circular scaffolds could also be wrapped around the anastomosed area of the lacerated tendons with the aiming to reduce tendon adhesions [10].

Therefore, we combined the gel and electrospinning technologies to produce a novel three dimensional hybridized collagen implant which has aligned ultrastructure and is composed of micro and nano collagen fibers with the aiming to mimic the intact tendon architecture both in size and architecture. This technology is a cost effective, practical and effective method in producing tendon tissue engineered based collagen implants. After *in vitro* evaluations and performing quality control tests, we investigated its efficacy on acute large Achilles tendon defect model in rabbits. Our hypothesis was that the tridimensional collagen implant probably motivates the inflammation, increases the rate of the healing response and guides the regenerated tissue in the injured area and possibly results in formation of lower peri-tendinous adhesion [10,13]. The architecture of the implant possibly aligns the newly regenerated collagen fibers through their designed orientation [10,24]. Possibly, the collagen implant is gradually absorbed and the newly regenerated tissue is substituted and then matures [10,25]. These criteria are possibly responsible in establishing the continuity of the tendon in the defect area and improving the functional performance of the injured tendon [24-29].

## Methods

### Ethics

All animals received human care in compliance with the Guide for Care and use of Laboratory Animals published by the National Institutes of Health (NIH publication No. 85–23, revised 1985). The study was approved by the local Ethics Committee of our research center.

### Preparation of the collagen implant

Collagen type I was extracted from the bovine superficial digital flexor tendon according to the methods of Foltran et al. [30]. The purity of the type I collagen was assessed by SDS/PAGE using 6% separating gels. The gel was then stained overnight with 0.017% (w/v) Coomassie blue R-250 (Bio-Rad®, Hercules, USA) in 38.8% methanol and 6.8% acetic acid. Subsequently, the gel was destained with 5% methanol and 5% acetic acid for 48 hours [30].

**Figure 1:**
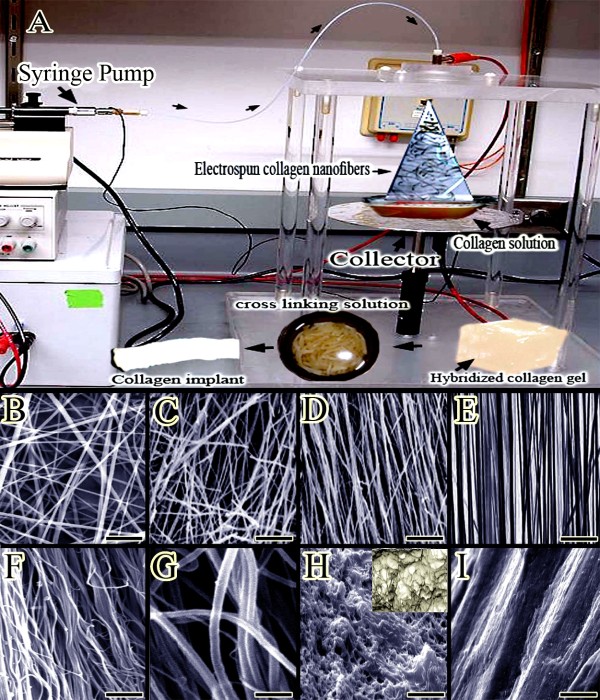
**Preparation of the collagen implant (Methods section: Preparation of the collagen implant).** (**A**) Preparation of the collagen implant. The collagen solution was placed in the syringe pump with the needle charged at 6 kV with respect to the collector. The nanofibers were harvested and mixed with fresh collagen solution incubated for final polymerization. This hybridized collagen gel was dried and cut into rectangular strips to form several prostheses which were cross-linked, sterilized and then dried. (**B,C**) Un-aligned electrospun collagen fibers constructed by electrospinning without dual plate device (SB = 1.7 μm). (**D**) Aligned electrospun collagen fibers were produced but the gap between the copper strips was 2 cm (SB = 2.2 μm). (**E**) Aligned electrospun collagen fibers were produced and the gap between the copper strips was 1 cm (SB = 2.2 μm). (**F**) Aligned internal architecture of the polymerized collagen fibers in the gel (SB = 15 μm). (**G**) The composition of the collagen implant at larger magnification (SB = 5 μm). (**H**) Transverse section of the collagen implant after crosslinking. Most of the implant is filled with the polymerized collagen fibers while little porosity is existed between these fibers (SB = 15 μm). (**I**) Surface of the collagen implant after cross linking (SB = 100 μm).

The electrospinning solution was prepared by mixing collagen (type I acid soluble from bovine tendon) in acetic acid (>99%; EMD, San Diego, CA). The solution (7.5% (w/w) collagen) was loaded into a syringe (Air-tite Products, Virginia Beach, VA) with a blunt-ended needle (18, gauge). The syringe was then placed on a syringe pump (New Era Pump Systems, Inc., NY) and directed toward a grounded collection device. Voltages of 6 kV were applied to the needle when the flow rate on the syringe pump was set to dispense at 0.15 mL/h. Uniformly aligned fibers were constructed by electrospinning onto a dual plate device. Dual plate devices used in this experiment consisted of two 2.5 × 0.5 cm copper strips attached to a gap substrate with Gluseal (Glustitch, Gulf Road Point Roberts, WA). The gap substrate was quartz glass (McMaster-Carr, Elmhurst, IL) with an electrical resistivity of 10^20^ Ωm. The gap between the copper strips was 1 cm, and each copper strip had a separate grounding wire. The dual plates were 4 cm away from the needle tip. The copper plates were removed after electrospinning [23]. Highly aligned and large electrospun collagen fibers with a diameter of 272 ± 183 nm were produced successfully (Figure 1A-E). The collagen matrix was then let to be dried at room temperature overnight to remove remaining solvents.

After electrospinning, acid-solubilized bovine tendon type I collagen was mixed with phosphate buffered saline (PBS) (Sigma, 10×) and NaOH (0.1 N) to prepare a collagen solution with pH between 7.2 and 7.4 and concentration of 10 mg/ml. This mixture was immediately pipetted into a syringe and injected into a custom made chamber (10 × 10 × 10 cm) box containing electrospun collagen matrix. The box was placed in an incubator at 4°C for 48 hours to polymerize the collagen gel and to produce large fibers (Figure 1). The collagens were aligned under 12 Tesla magnetic fields (CRETA, Grenoble) during polymerization [31]. The range of the fiber diameter was 1.82 μm to 3.19 μm. The electrospun collagen matrix acted as a core with its fibro-conductive characteristics and improved alignment of the newly formed collagen fibers. The collagen composite was cut into several pieces of the same size and shape as the rabbit’s Achilles apparatus (L = 2 cm, H = 3. 5 mm, W = 3 mm) (Figure 1A).

For cross linking, the implants were suspended in iso-osmolar 0.1% riboflavin solution with Dextrane T500 (as a photosensitizer), 20 minutes before the irradiation to allow sufficient saturation of the implant. The implants were irradiated for 60 minutes with UV (wavelength of 365 nm) at a working distance of 5 cm, with an irradiance of 3 mW/cm^2^ corresponding to a surface dose of 5.4 J/cm^2^, (UV-X™, Peschke Meditrade, Cham, Switzerland). During irradiation, drops of riboflavin solution were applied to the implant every 4 to 5 minutes to sustain the necessary concentration of the riboflavin. The implants were neutralized with distilled water four times (30 min each) [32].

The final product was repeatedly washed with distilled water to remove the residual enzymes and chemical reagents, let to be dried overnight, received 100 Gray g-radiation for 120 min and then suspended in ethanol 96% to produce and maintain its sterility until surgery. Two hours before surgery, the implant was dried to evaporate the ethanol residues and repeatedly washed with distilled water (10 min × 12 times) and finally suspended in isotonic saline solution to soften the implant for better surgical handling [33].

### Initial tests of the scaffold

#### Scanning morphology of the scaffolds

Morphology of the scaffolds (*n* = *10* scaffold) was studied by scanning electron microscopy (SEM). For calculating the proportion of fiber alignment, 100 fiber was counted in each photomicrograph (*n* = *100*) and the proportion of the aligned fibers/randomly oriented fibers were reported as Mean and SD. Density of the collagen fibers per photomicrographs was calculated by computer software (Image J, NIH, NY, USA). Porosity (free spaces between the collagen fibers) was defined as the ratio of empty area to the total area and the mean and standard deviation (SD) of 100 photomicrographs (*n* = *10* for each specimen) was reported. Transverse diameter of the 100 collagen fibers and 100 pores were measured using software scaling system (Photoshop CS-5 extended final, Adobe Co. CA, USA) and then the mean and SD of the measured values was reported [23].

#### *In vitro* collagenase degradation test

The biological stability of the cross-linked collagen implant was evaluated by *in vitro* collagenase biodegradation test. The scaffolds were incubated in phosphate buffered saline (PBS, pH 7.4) containing a given concentration of collagenase (type I, Sigma) at 37°C. The degradation was terminated at the given time interval by incubating the assay mixture in an ice bath immediately. Following centrifugation at 1000 rpm for 10 min, the clear supernatant was hydrolyzed with 6 M HCl at 120°C for 12 h. The amount of hydroxyproline released from the collagen molecules in the scaffolds was measured by absorbance spectroscopy (CARY 100 BIO, USA) at the wavelength of 560 nm. The biodegradation degree is defined as the percentage of the released hydroxyproline from the scaffolds to the completely degraded one with the same composition and weight. We compared the collagen implants which were cross-linked by UV + riboflavin with the collagen implants which were cross-linked by dehydrothermal (DHT) crosslinking method. The DHT scaffolds were dehydrated at 105°C under vacuum (less than 0.2 mbar, DHT) for 24 h.

#### Mechanical testing of the scaffolds

The un-cross-linked collagen implants and the cross linked collagen implants were subjected to the mechanical testing using the tensile INSTRON machine (INSTRON, London, UK). Scaffolds were hydrated for 24 h in PBS (pH 7.2) and drawn at a speed of 10 mm/min. The maximum load, maximum stress, strain and modulus of elasticity of the implants were extracted from the generated force-displacement and stress–strain curves.

#### Microbiological tests

The scaffolds were tested for sterility immediately after sterilization so that they were immersed in a Nutrient Agar Broth (NEOGEN Co. Lansing, MI, USA) to cultivate fastidious micro-organisms and maintained under agitation at 25°C for 48 h. Non-sterile scaffolds (*n* = *10*) were used as negative control while the Graft jacket (Regenerative Tissue Matrix, Wright Medical Technology, Inc. San Antonio, TX, USA) was used as the positive control (*n* = *10*). Bioburden Challenge Test was also performed in a following manner: Test organisms that included Methicillin-resistant *Staphylococcus aureus* (MRSA; DMST 20645 lot no. 3273, NIH) and *Bacillus subtilis* (ATCC 6633 DMST 15896 lot no. 3479, NIH) were suspended in tryptic soy broth (TSB) (Sigma-Aldrich Co. LLC) to provide a final concentration of 10^2^ and 10^4^ cfu/mL. Inoculation of the test carrier was performed by using a micropipette to place 20 μL of the test suspension on the surface of scaffolds and left to dry in the incubator for 18 h at 37°C. The scaffolds were then sterilized as described before. After sterilization, the scaffolds were then transferred to test tubes containing TSB and were incubated at 25°C for 7 days. The turbidity of the TSB was measured every day using a UV spectrophotometer at 625 nm wavelength and using McFarland Standards as a reference to estimate the number of colonies [34].

#### Determination of endotoxin content

The Limulus Amebocyte Lysate (LAL) (Toxin Sensor^TM^ Gel Clot Endotoxin Assay Kit, Gen Script Inc. Piscataway, NJ, USA) test was used according to the manufacturer’s recommendation. The LAL reagent was mixed with the samples into the endotoxin-free vials, and then incubated at 37°C for 60 minutes. Each vial was then inverted and checked whether a gel was formed or not.

#### Cell seeding

Rat skin fibroblasts (cell line CRL-1213) were obtained from the American Type Culture Collection (Manassas, VA) and cultured (37°C, CO_2_ 5%, pH = 7.4) in Dulbecco’s Modified Eagle Medium supplemented with 10% fetal bovine serum, 20 U/ml penicillin, and 20 μg/ml streptomycin (Invitrogen, Carlsbad, CA). Cell culture medium was replaced every three days. The cells were passaged at confluence and the 4–8th passage fibroblasts were used for the seeding. A conventional static seeding method was used to seed cells onto the scaffolds. Briefly, 50 μL cell suspension containing about 5 × 10^5^ cells was cultivated on every collagen scaffold and incubated for 1.5 h, allowing the cells to attach to the collagen implant under standard culture conditions (37°C, in a humidified atmosphere containing 5% CO_2_ and 95% air). The remaining 950 μL media was then added to the wells. After 24 h, the non-adhering cells were washed off with PBS for three times and the samples were transferred to new tissue culture plates. Numbers of the cells remaining in the collagen scaffold, in the culture media, and on the tissue culture plate wells were measured by MTS (3-(4,5-dimethylthiazol-2-yl)-5-(3-carboxymethoxyphenyl)-2-(4-sulfophenyl)-2H-tetrazolium) assay (MTS-kit, bioassay; Promega). The seeding efficiency was calculated according to equation: Seeding efficiency (%): number of cells remaining in the matrix/number of cells seeded × 100.

#### Cell culture, MTS and live/dead cell assays

The cell-seeded scaffolds experienced a 20-day static culture. In the culture period, the media were first replenished after the first 7 days and then every 3 days. During the culture period, MTS and live/dead cell assays were performed on the samples at three time points (5, 10, and 20 days). For the MTS assay; the cultured scaffolds were rinsed with PBS to remove non-adhering cells and were transferred to new wells containing 300 μL of phenol-red–free RPMI media (Roswell Park Memorial Institute [RPMI]; Invitrogen) and 100 μL of MTS-PMS in PBS solution (0.046 mg/ml PMS (Phenazine Methosulphate, PMS; Aldrich) and 2 mg/ml MTS).

The samples were then incubated in a 37°C incubator for 1 h, after which 100 μL of the solution was withdrawn to measure the absorption at 490 nm on a microplate reader. Number of cells were quantified by comparing the absorbance of a series of known numbers of viable cells [35]. Cell viability was determined by live/dead cell assay using fluorescein diacetate (FDA, Molecular Probes, Invitrogen Corporation) (live) and propidium iodide (Cayman Chemical Company, Michigan, USA) (dead). Briefly, the cultured scaffolds were rinsed with PBS to remove non-adhering cells and were incubated in appropriate amounts of fluorescent dye for 45 min at 37°C. The scaffolds with the fluorescence stained cells were viewed under a Nikon fluorescent microscope [36].

Two parameters were measured using live/dead cells assay. First, the percentage of live cells was measured. Ten randomly-chosen fields of view were photographed from each collagen implant (a total of 10 implant yielded 100 photos per material). The cells were counted. N _Live_ is the number of live cells, and N _Dead_ is the number of dead cells. The percentage of live cells, P _Live =_ N _Live_ / (N _Live_ + N _Dead_). The second parameter was cell attachment, C _Attach_. It is the number of live cells attached on the specimen divided by the area, A: C _Attach_ = N _Live_ / A. Both P _Live_ and C _Attach_ were measured, because a high value of P _Live_ only means that there are few dead cells; it does not necessarily mean a large number of live cells that are attached to the specimens. C _Attach_ quantifies the absolute number of live cells anchored on the collagen implant per surface area [37].

#### Morphology of the fibroblast-seeded scaffold

The tissue constructs were removed from cell culture after 5, 10 and 20 days, and after routine histologic preparation were stained with hematoxylin and eosin and examined by a light microscope (Olympus, Tokyo, Japan) for regular cell counting and studying their distribution in the histopathologic fields. Number of cells per microscopic field in five fields per specimen (*n* = *10*) were counted. At 5, 10 and 20 days after seeding, the culture was discontinued and the scaffolds were washed with PBS and fixed with 2.5% glutaraldehyde at room temperature for 24 h. After being washed with PBS to remove the remaining glutaraldehyde, the scaffolds were dehydrated with a graded series of ethanol. The scaffolds were further dehydrated with acetone and treated with isoamylacetate. After being dried by the critical point dry method, the scaffolds were coated with an ultrathin gold layer and observed by scanning electron microscopy (SEM, Cambridge. London, UK).

#### Measurement of the scaffold contraction

The diameters of the seeded and unseeded scaffolds were determined with a digital caliper (n = 10). Contraction of the scaffolds was calculated by dividing the diameter change of each scaffold at the termination of the culture time by its original diameter. Cell-mediated contraction (CMC) was determined by subtracting the contraction of the unseeded scaffolds from the contraction of the seeded scaffolds.

CMC = (original diameter-diameter/original diameter) _seeded_ – (original diameter-diameter/original diameter) _unseeded._

### *In vivo* experiment

#### Animals and grouping details

Forty skeletally-mature male White New Zealand rabbits of 12 ± 2 months age and 3.21 ± 0.17 kg body weight were randomly divided into treatment (*n* = *20*) and control (*n* = *20*) groups. Another 35 rabbits were divided into 7 equal subgroups, to study the tissue reactions in the injured area at macroscopic and histopathological levels, and the mechanism of degradation of the collagen implant after its incorporation into the tissue defect.

The left Achilles tendon of each animal in both groups was designated as the transected/injured tendon and the right contra-lateral tendon (NCT) was left intact. The animals were housed in individual standard rabbit cages and maintained on a standard rabbit diet, with no limitation of access to food or water.

The left Achilles tendon of the treatment group was compared with their right NCT and also the untreated, injured control tendons (ICTs) at 120 days post injury (DPI). They were additionally compared with those of the pilot groups at 7, 10, 15, 20, 30, 40 and 60 DPI, respectively.

#### Premedication and anesthesia

Premedication was performed by intramuscular injection of 1 mg/kg acepromazine maleate. The animals were anesthetized by intramuscular injection of 30 mg/kg Ketamine combined with 0.05 mg/kg Xylazin hydrochloride (Neurotranq 1%; Ketamin 10%; Xylasin 2%, all from Alfasan Co., Woerden, Netherlands) [24].

#### Injury induction and surgical reconstruction

**Figure 2:**
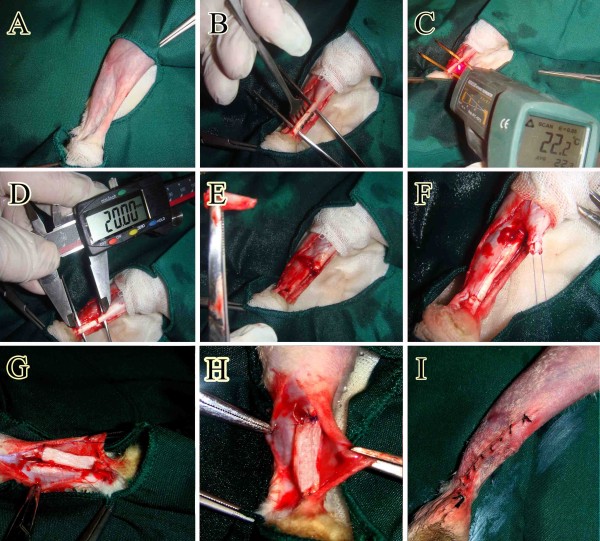
**Surgical intervention and implantation of the collagen prosthesis (Methods section: Injury induction and surgical reconstruction).** Plantar part of the Achilles tendon was prepared aseptically (**A**). After skin incision the left Achilles structure was exposed (**B**). The temperature of the surface of the Achilles tendon was measured by a digital laser thermometer (**C**). Two cm of the Achilles tendon between the gastrocnemius and calcaneus was measured using digital caliper (**D**). This part of the tendon was surgically cut and discarded (**E**). The remnants of the Achilles structures were maintained in their normal anatomical direction by application of the Kessler suture (**F**). The collagen implant was then implanted in the defect area and the suture was led to pass throughout the implant (**G&H**). The skin over the lesion was closed in a simple interrupted manner, with low tension on the sutures (**I**).

The left hind limb was shaved and prepared for aseptic surgery (Figure 2A). After making a lateral longitudinal incision on the skin over the left Achilles tendon, a skin flap was reflected medially to expose the Achilles tendon (Figure 2B,C). Two cm of the Achilles tendon with its paratenon was excised, from approximately 0.5 cm distal to the gastrocnemius muscle to 0.5 cm proximal to the calcaneal tuberosity (Figure 2D,E).

Primary realignment of the tendon extremities was undertaken using double stranded monofilament absorbable polydioxanon 0–4 suture material and a straight taper-cut orthopedic needle (PDS, Ethicon, INC.1997, Johnson & Johnson, USA), in a modified Kessler core pattern, to arrange the remaining tendon edges according to the normal anatomical orientation but leaving a 2 cm gap between the extremities (Figure 2F) [26,27]. This method was applied to all groups including the control, treatment and pilots. In the treated and the pilot animals the double-stranded sutures were routed through the longitudinal axis of the prosthetic implants with the suture knots at the latero-distal extremity of the reconstructed Achilles tendons (Figure 2G,H). The skin over the lesion was closed using 2–0 silk continuous pattern (Figure 2I).

#### Clinical examinations

Based on the methods of Oryan et al. [25] the transverse diameter of the injured area, including the injured tendon, subcutaneous area and skin, were measured, using a micrometer measurement device (Guanglu electronic digital caliper, Anyang, South Korea), before injury, and then weekly until the end of the experiment. The surface temperature of the injured area and the NCT was measured with a laser heat-detector device (Mastech, MS6530 Infrared Thermometer, Seoul, South Korea) [28,29].

Lameness and comfortable/uncomfortable physical activities were monitored at weekly intervals and defined and scored according to the methods of Oryan et al. [29]. The weekly scores during the course of the experiment were added together and total scores have been expressed as median (minimum – maximum) (Table 1).

**Table 1:** Base scoring system used for determination of lameness, and pain in rabbits after tendon transection

***1. The tarsal flexion degree of the injured leg compared to the normal leg, both in the cage and on the floor***					
**Score**	Between legs		Estimation of the degree		Condition
**0**	equal		75-90		normal
**1**	non equal		50-74		mild
**2**	non equal		30-50		moderate
**3**	non equal		>30		severe
**4**	non equal		<15		extensively severe
***2. Weight distribution of each animal on the hind limbs, in the cage and on the floor***					
	Weight distribution between limbs	Weight distribution between hind legs	the most weight bearing legs	The injured left hind leg condition	
**0**	mostly hind limb	equal	both hind limbs	weight bearing	normal
**1**	mostly hind limb	not equal	right hind limb	weight bearing	mild
**2**	mostly forelimbs	not equal	both forelimbs & right normal hind limb	weight bearing	moderate
**3**	not Equal	not equal	both forelimbs and right normal hind limb	non weight bearing	severe
**4**	not Equal	not equal	non weight bearing (sternal recumbency)	non weight bearing	extremely sever
***3. Pain in palpation of the injured area and pain in complete extension of the injured leg***					
	description				
**0**	no reaction				normal
**1**	occasional vocalization				mild
**2**	frequent vocalization				moderate
**3**	vociferous vocalization, withdraw limb, bites, struggles				severe
***4. Heel and toe position of the injured leg (left hind paw)***					
	Heel	Toe			Condition
**0**	up	down			normal
**1**	Near the floor (up)	down			mild
**2**	down	down			moderate
**3**	down	up			severe
***5. Swelling at the injured area (left hind paw)***					
	Swelling (injured area)				
**0**	is not tender, warm and bowed				normal
**1**	slightly warm and bowed, color is not changed				mild
**2**	tenderness, bowed and completely warm. color in not changed				moderate
**3**	obvious tenderness, bowed and warm. color changed.				severe

#### Bioelectrical characteristics

To determine the Direct Transmission Electrical Current (DTEC; microamp) and the Tissue Resistance to Direct Electrical Current (TRDEC; micro-ohm) of the ITTs, ICTs and their NCTs, a bioelectrical measurement device (MASTECH, Digital multi pen type meter, Seoul, Korea) was used. The negative probe was placed on the medial part of the tendon and the positive probe was placed at lateral side. These characteristics were analyzed at weekly intervals, (from 0 to 120 DPI).

The ohm (*Ω*) is defined as a resistance between two points of a conductor when a constant potential difference of 1 volt, applied to these points, produces in the conductor a current of 1 ampere, the conductor not being the seat of any electromotive force. The ampere (SI unit symbol: A), often shortened to amp, is the SI unit of electric current (quantity symbol: *I*,*i*) and is one of the seven SI base units. In practical terms, the ampere is a measure of the amount of electric charge passing a point in an electric circuit per unit time with 6.241 × 10^18^ electrons, or one coulomb per second constituting one ampere.

#### Hematological parameters

Immediately before euthanasia, blood sample (1 cc/animal) was collected from each animal, transferred into the EDTA tube and indirectly tested for different cell typing using standard clinical cell-counter (Veterinary Auto-analyzer, Cambridge, UK).

#### Euthanasia

The animals were euthanized by intra-cardiac injection of a combination of 35 mg/kg Ketamine, 2 mg/kg Xylazine, and 1 mg/kg Acepromazine maleate (All from Alfasan Co., Woerden, Netherlands) with 2 mg/kg gallamine triethiodide (Specia Co., Paris, France) [25,27,29,38].

#### Sample collection

In the pilot groups (*n* = *5* for each), the ITTs and their NCTs were dissected and assessed for gross morphology and histopathological observations. The left and right Achilles tendons of the animals of the treated (*n left* = *20*, *n right* = *20*) and control groups (*n left* = *20*, *n right* = *20*) were harvested at 120 DPI. After they were evaluated at gross pathological level, half of the ITTs, and NCTs of the treated group and ICTs and NCTs of the control group, were used for determination of the dry matter content, water uptake and water delivery analysis. The rest half of the samples were prepared for histopathological observation (*n left* = *10*, *n right* = *10 for each group*).

#### Determination of water uptake, water delivery and percentage dry weight

For percentage dry weight analysis, the ITTs or ICTs and their NCTs were weighed immediately after euthanasia, freeze-dried to a constant dry weight, and the percentage dry weight was then calculated [25,27,29]. To determine the water uptake capacity each fully dehydrated collagen implant or tendon was immersed in 0.9% isotonic saline solution, at 37°C, for 48 hours. To gain its final wet weight. This weight was used as an index of wet weight of the implant, which was then freeze-dried. The fully dehydrated material was weighed and then immersed in 0.9% isotonic saline solutions at 37°C. From minute 1 to minute 1920 after immersion the samples was weighed at various time points. The water uptake capacity was calculated using the following equation: Index of water uptake = weight (dry) /time. The time refers to the point that the samples gained their maximum wet weight.

For calculation of their water delivery capacity, the fully hydrated samples, were placed in a dry place, at 37°C, and left to evaporate their water content. From minute 1 to minute 3840 after air exposure, the samples were weighed at various time points. The index of delivery was calculated using the following equation: Index of water delivery = (Wwet)/Wdry × 100/time. The time refers to the point that the samples reached to their dry weight. Analysis was performed based on the indices of normal tendons.

#### Gross and histopathology

At the gross pathological level, each injured or its NCT was evaluated for color changes, adhesion to the surrounding tissues and any other abnormalities such as tendon degeneration, muscle fibrosis and atrophy. Longitudinal tendon samples from injured and normal tendons of all 10 animals in each group were collected for histopathology. After fixation in 10% neutral buffered formalin, the tendon samples were washed, dehydrated, cleared, embedded in paraffin wax, sectioned longitudinally at 4–5 μm thickness, stained with hematoxylin and eosin and examined by light microscopy (Olympus, Tokyo, Japan). Gross appearance and histology of the tissue samples were used to define the mechanistic effects of the bioimplant on tendon healing. We also tried to evaluate the mechanisms of tissue reaction in response to the collagen implant, biodegradability and biocompatibility of the collagen implant in the defect area. Three veterinary pathologists, who were expert in the field of tendon healing, observed the samples and reported their observations and opinions. The conclusion of their reports was mentioned in the Results section. The quantitative results are not shown [24,28].

#### Statistical analysis

After application of the normal distribution test, the measured values of the injured tendons of each group were compared statistically with the measured values of their NCTs of the same group, using paired-sample *t*-*Test*. The measured values of the right and left tendons of the treated animals were compared with those of the control animals using the independent sample *t*-*Test*. Mann–Whitney U Test was performed to statistically analyze the base-scoring system described in the methods (Table 1). Statistics were performed using the computer software SPSS version 17 for windows (SPSS Inc., Chicago, IL, USA). Differences of *P* < *0*.*05* were considered significant. For base scoring data the median and range of the scores was reported in the results section [median (min-max)] [25,27-29].

## Results

### In vitro results

#### Collagen purity

**Figure 3:**
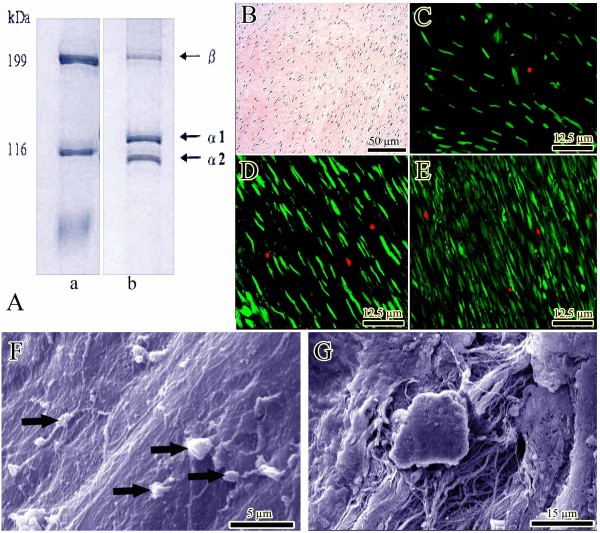
***In vitro *tests (Results section: Initial tests of the scaffold).** (**A**) SDS-PAGE images of type I collagen using 6% poly-acrylamide gel to examine the purification of collagen extracts. Lane a: molecular marker, lane b: bovine collagen. (**B**) Histologic section of the constructs after 20 days of cell culture. The proliferating fibroblasts are infiltrated to the implant and proliferated. (**C-E**) Cell viability was determined by live/dead cell assay using fluorescein diacetate (live) and propidium iodide (dead). (**C-E**) shows day 5, 10 and 20 after cell seeding. Almost all of the fibroblasts are green, indicating the cells are live. The lack of Propidium iodide stained dead cells (red) supports the idea that normal rat fibroblasts have attached to the scaffold and that the majority of the cells are viable. (**F&G**) Surface and inside of the collagen implant after 20 days of cell seeding, respectively. The cells proliferated (arrows) and produced matrix.

Collagen extract contained two main α- chains (α_1_ and α_2_) and one β chain with very little contamination of other proteins. This suggested that collagen type I extract were pure with very little protein degradation product or other protein contamination (Figure 3A).

#### Morphology of the collagen implant

The transverse diameter of the electrospun fibers in the 2D collagen matrix was 272 ± 183 nm (ranging 89 to 455 nm) (Figure 1E). We used both the ground plate and rotating mandrel (750–5500 rpm) to collect the electrospun collagen fibers and served them as the control for dual plate device which we used to produce aligned collagen nanofibers. The collagen fibers were homogenously collected by the dual plate device so that they were highly aligned in the electrospun sheet (Figure 1E). The proportion of the aligned collagen fibers/randomly formed collagen fibers was 95.28 ± 2.37/ 4.72 ± 0.47 (Figure 1E). The low standard deviation of the collagen fibers suggest that the collagen fibers were highly aligned. In contrast the collected electrospun fibers on the ground plate showed no alignment and the collagen fibers were randomly distributed at different directions of the matrix (Figure 1B and C). The highest alignment degree of the electrospun collagen fibers was seen in the rotating mandrel method with the 5500 rpm so that the proportion of the aligned collagen fibers/randomly collagen fibers was 73.32 ± 12.38 / 26.58 ± 6.77 which was significantly lower than the alignment proportion which was measured for the dual plate device method (*P* = *0*.*001*). The thickness of the electrospun collagen scaffold was 280 μm. The density of the electrospun collagen fibers was 92.38 ± 2.99% (Figure 1E).

The transverse diameter of the randomly polymerized collagen fibers in the gel were 2.86 ± 0.54 μm which was not significantly different as compared with the collagen fibers of the hybridized collagen implant (polymerized collagen fibers + electrospun collagen fibers). The transverse diameter of the polymerized microfibers in the collagen gel (3D) was 2.54 ± 0.68 μm (ranging 1.82 to 3.19 μm) (Figure 1G). The hybridized collagen implant showed a combination of nano and micro collagen fibers which were not seen in the randomly polymerized collagen gel (Figure 1G). The density of the collagen fibers in the randomly polymerized collagen gel was 47.82 ± 8.31% whereas the density of the collagen fibers in the hybridized collagen gel (implant) was 82.38 ± 3.15% which means combination of the electrospun nanofibers with polymerized collagen fibers of the gel system, significantly increased the density of the collagen fibers (*P* = *0*.*001*) as compared with the randomly polymerized collagen fibers (Figure 1F). This method of combination, also significantly increased the alignment of the collagen fibers in the hybridized gel as compared with the randomly polymerized collagen gel (*P* = *0*.*001*). The proportion of the aligned collagen fibers/randomly formed collagen fibers in the hybridized collagen implant (Figure 1F) was 71.27 ± 12.81/28.91 ± 4.22 while this value for the randomly polymerized collagen gel was 22.81 ± 6.02/77.19 ± 14.21. In cross sections of the collagen implant, the porosity was 17.62 ± 4.88% (Figure 1H). The average diameter of these pores was 8.43 ± 4.27 μm (*n* = *100* pores).

#### *In vitro* collagenase degradation

The cross linking method used in this study was even more resistant than the DHT method because the DHT cross-linked scaffolds were fully (100%) degraded after incubation in the collagenase solution for 12 h while only 18.38 ± 6.44% of the UV cross-linked collagen implant was degraded at that time.

#### Mechanical characteristics of the scaffolds

The maximum load ^N^ (28.33 ± 2.19 ^cross-linked collagen^*vs*. 4.56 ± 1.92 ^uncross-linked collagen^, *P* = *0*.*001*), maximum stress ^N/mm2^ (2.69 ± 0.47 ^cross-linked collagen^*vs*. 0.40 ± 0.11^uncross-linked collagen^, *P* = *0*.*001* for all), and modulus of elasticity ^KPa^ (43.81 ± 4.19 ^cross-linked collagen^*vs*. 4.05 ± 1.28 ^uncross-linked collagen^) of the cross-linked collagen implant (*n* = *10*) were significantly higher than those of the un-cross-linked (*n* = *10*) collagen implants. Also the maximum strain^%^ of the cross-linked collagen implant (*n* = *10*) was significantly lower than those of the un-cross-linked (*n* = *10*) collagen implants (61.34 ± 4.71 ^cross-linked collagen^*vs*. 98.55 ± 7.23 ^uncross-linked collagen^, *P* = *0*.*001*).

#### Sterility of the scaffolds

After 48 h incubation, the untreated controls (unsterilized scaffolds) produced signs of growth during sterility test whereas the Graftjacket and the sterilized scaffolds remained free of growth throughout the same time period and showed successful sterilization throughout the treatment durations. The result of bioburden test also showed that the sterilization technique was effective because the broth media was clear after challenging with high bacterial suspension even after 7 days incubation.

#### Endotoxin level of the scaffolds

The endotoxin levels of the collagen implants were below 0.25 EU/ml and no gel or turbidity was formed.

#### Seeding efficacy, cell number, cell density, cell viability and cell proliferation

After 24 h, 78.01% of the fibroblasts remained on the collagen scaffold while 12.84% existed in the well and 9.15% of fibroblasts remained on the media. Within the 20 days of the culture period, the number of fibroblasts continuously increased in the collagen scaffold. A number of the proliferated cells in the scaffolds were 89.32 ± 12.84 (day 5), 202.17 ± 31.09 (day 10), and 358.92 ± 66.82 (day 20), at histology level (×200, Figure 3B). Number of attached cells per 1 mm area of the scaffold was 365.28 ± 23.71 (day 5), 632.81 ± 38.92 (day 10), and 1071.09 ± 93.25 (day 20). Number of cells which was obtained from MTS assay was 9.16 × 10 ^6^ (day 5), 21.05 × 10^6^ (day 10) and 34.82 × 10^6^ (day 20). The results of the Live/Dead cell Assay showed that almost all of the fibroblasts were green, indicating the cells were live (Figure 3C-E). Lack of propidium iodide (red) stained dead cells supports the idea that normal rat fibroblasts were attached to the scaffold and that the majority of the cells were viable (Figure 3C-E). The number of FDA stained viable cells in the scaffold (×200) was 84.71 ± 9.41 (day 5), 186.33 ± 27.91 (day 10), and 321.34 ± 49.23 (day 20) and the percentage of viable cells was 94.83% (day 5), 92.16% (day 10) and 89.52% (day 20) of the total cellularity (vitality index). The pattern of cell proliferation was homogenous in the scaffold (Figure 3B-E). The SEM images indicated that the fibroblasts well proliferated both on the surface (Figure 3F) and internal architecture (Figure 3G) of the implants and produced matrix.

#### Scaffold contraction

Five, 10, and 20 days after cell culture, The CMC contraction of the cross linked scaffolds was significantly lower than the non-cross-linked scaffolds (0.04 ± 0.01^day 5^, 0.05 ± 0.01^day 10^, 0.07 ± 0.01 ^day 20^, _cross-linked scaffold_ vs. 0.34 ± 0.02 ^day 5^, 0.51 ± 0.04 ^day 10^, 0.67 ± 0.05 ^day 20^, _uncross-linked scaffold_). Twenty days after cell culture, the CMC value was 0.67 meaning that the scaffold had been largely contracted. In contrast to the non-cross-linked collagen implant, no obvious contraction was observed for the cross-linked collagen implant during the 20 days. These results suggest that the cross linking method was effective to preserve the architecture of the collagen implant at different stages of cell culture.

### *In vivo* results

#### Clinical examinations

During the course of the experiment, the treated animals showed significantly better scoring for the tarsal flexion degree (39 (31–44) *vs*. 50.5 (40–59), *P* = *0*.*001*), weight distribution on their legs (45.5 (35–57) *vs*. 56.5 (46–60), *P* = *0*.*001*), pain on palpation of the injured area (37 (32–41) *vs*. 45 (39–48), *P* = *0*.*001*), heel and toe position (34.5 (31–38) *vs*. 46 (38–48), *P* = *0*.*001*), and swelling at the injured area (40 (36–45) *vs*. 47 (44–48), *P* = *0*.*001*), compared to the control animals. Therefore, the treated animals had a better weight bearing and physical activity compared to the control animals. Also their swelling following injury subsided faster and they showed less pain after palpation of the injured area, compared to those of the control animals.

**Figure 4:**
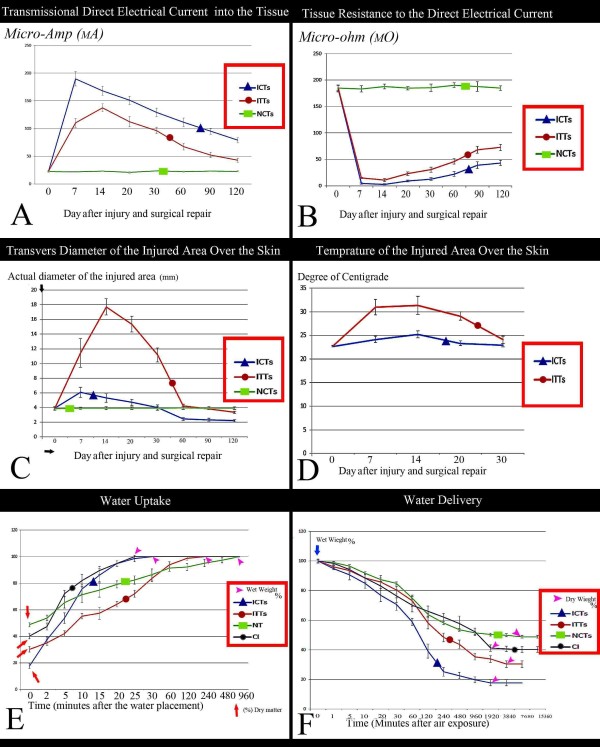
**Clinical, bioelectrical and chemical diagrams (Results section: Dry matter content, water uptake and water delivery). A**: Transmission direct electrical current of the tissue. Compared to ITTs the pattern of the ITTs is closer to the normal tendon. **B**: Tissue resistance to direct electrical current. The pattern of the ITTs is closer to the normal tendon. **C**: Diameter of the injured area from the skin over the lesion. The ITTs showed a significantly greater diameter at 7 and 14 DPI compared to the ICTs (P = 0.001). After 21 days until the end of the experiment the transverse diameter of the ITTs was reduced. At 120 DPI, the transverse diameter of the ITTs was significantly higher than the ICTs (P = 0. 001) but no significant differences were seen between the ITTs and the intact tendons at this stage. **D**: Surface temperature of the injured area. Treatment significantly increased the temperature of the ITTs compared to the ICTs but this increase was subsided and reduced to normal value after, 30 days of injury. **E&F**: Water uptake and Water delivery characteristics of the ITTs, ICTs, normal tendons, and collagen implant. Note the similarity pattern between the diagrams of the ITTs and the normal contralateral tendons. The ICTs shows a different pattern compared to the ITTs and the intact tendons.

The surface temperature of the injured area of the treated animals significantly increased at 7th DPI compared to those in the 0 DPI, (30.99 ± 1.63°C *vs*. 22.74 ± 0.18°C, *P* = *0*.*001*). However, there were no significant differences in the temperature of the injured area of the control animals between days 0 (before surgery) and 7 after injury (22.68 ± 0.16°C *vs*. 24.14 ± 0.68°C, *P* = *0*.*119*). The temperature of the injured area of the treated animals was high during the first three weeks after injury and then gradually subsided to normal value at 30 DPI (24.09 ± 0.79°C *vs*. 22.93 ± 0.22°C, *P* = *0*.*001*) so that a significant decrease was seen at this stage compared to the 7, 14, and 21 DPI (24.09 ± 0.79°C *vs*. 29.09 ± 0.83°C, 31.34 ± 1.88°C, 30.99 ± 1.63, *P* = *0*.*001*) (Figure 4D). Unlike treated animals, the temperature of the injured area of the control animals showed a significant increase 14 DPI and then subsided to normal value at 20 DPI so that there were no significant differences between the temperature of the ICTs at 0 and 20 DPI (*P* = *0*.*055*). The highest temperature of the injured area was recorded at 14 DPI in both groups but the temperature of the ITTs was significantly higher compared to the ICTs (31.34 ± 1.88°C *vs*. 25.18 ± 0.73°C, *P* = *0*.*001*) (Figure 4D).

The transverse diameter of the injured area from the skin over the lesion showed a significant increase in both groups at 7 and 14 DPI (*P* = *0*.*001*). However, the measured values were significantly higher in the ITTs compared to the ICTs (Day 7: 11.43 ± 1.96 mm *vs*. 6.07 ± 0.7 mm, *P* = *0*.*001*; Day 14: 17.71 ± 1.13 mm *vs*. 5.32 ± 0.59 mm, *P* = *0*.*001*). In the ITTs, an increase in the diameter of the injured area continued for 14 days but in the ICTs the increase lasted up to 7 DPI (Figure 4C). The transverse diameter of the injured area of the ITTs reduced to normal value at 60 DPI so that there were no significant differences between these values at 0, 60 and 90 DPI (*P* > *0*.*05*). Reduction in the diameter of the injured area continued to 120th day after injury so that the transverse diameter of the injured area at 120 DPI was significantly lower than 0 DPI (3.36 ± 0.16 mm *vs*. 3.86 ± 0.22 mm, *P* = *0*.*001*). A similar pattern was seen in the ICTs, however, in comparison between the two groups, the transverse diameter of the injured area in the ICTs was significantly lower than the ITTs at 60, 90 and 120 DPI (2.44 ± 0.21 mm *vs*. 4.24 ± 0.23 mm, *P* = *0*.*001*; 2.34 ± 0.19 mm *vs*. 3.83 ± 0.09 mm, *P* = *0*.*001*; 2.22 ± 0.15 mm *vs*. 3.36 ± 0.16 mm, *P* = *0*.*001* respectively). Also the transverse diameter of the ICTs at 120 DPI was significantly lower than 0 DPI (2.22 ± 0.15 mm *vs*. 3.92 ± 0.22 mm, *P* = *0*.*001*) (Figure 4C).

#### Hematological findings

At 120 DPI, there were no significant differences between the number of red blood cells (6.48 ± 0.84 × 10^12^/L *vs*. 6.41 ± 0.73 × 10^12^/L, *P* = *0*.*919*), Leukocytes (8425.14 ± 336.68 × 10^9^/L *vs*. 8072.28 ± 681.48 × 10^9^/L, *P* = *0*.*875*), Lymphocytes (35.00 ± 2.26% *vs*. 37.00 ± 1.49%, *P* = *0*.*924*) and platelets (338.28 ± 36.21 × 10^9^/L *vs*. 362.00 ± 27.21 × 10^9^/L, *P* = *0*.*644*) of the peripheral blood of the treated and control animals. However, the proportion of the neutrophils was lower in the treated animals compared to the control animals at this stage (60.42 ± 2.22% *vs*. 66.85 ± 1.77%, *P* = *0*.*001*) but in comparison between the proportion of the neutrophil/lymphocyte with normal range, no signs of neutropenia were observed in the treated and control groups.

#### Bioelectrical characteristics

There were no significant differences between the direct transmission electrical current (DTEC or micro-amp) of the ITTs and ICTs just before the surgical procedure (*P* = *0*.*968*, Day 0). However, the DTEC of the ICTs significantly increased at the 7th DPI (189.72 ± 13 ^micro-amp (μA)^*vs*. 22.24 ± 1.01 ^μA^, *P* = *0*.*001*) compared to day 0 after injury and then significantly decreased until at the end of the experiment (days 7 *vs*. 14 *vs*. 20 *vs*. 30 *vs*. 60 *vs*. 90 *vs*. 120 after injury, *P* = *0*.*001* for all comparisons). At 120th DPI, the DTEC of the ICTs was significantly higher than the 0 DPI (79.16 ± 4.13 ^μA^*vs*. 22.24 ± 1.01 ^μA^, *P* = *0*.*001*). The DTEC of the ITTs significantly increased at 7 and 14 DPI with the highest level on 14th DPI (22.75 ± 0.82 ^μA^ (day 0) *vs*. 110.12 ± 7.98 ^μA^ (Day 7) *vs*. 137.46 ± 7.05 ^μA^ (Day 14), *P* = *0*.*001*) and then it significantly decreased at weekly intervals until the end of the experiment (*P* < *0*.*05*). The highest level of the DTEC of the ITTs (observed on 14th DPI) was significantly lower than the highest level of the ICTs (observed at 7th DPI) (137.46 ± 7.05 ^μA^*vs*. 189.72 ± 13 ^μA^, *P* = *0*.*001*). Although the DTEC level of the ITTs was significantly higher at 120th DPI than 0 DPI (42.82 ± 3.61 ^μA^*vs*. 22.75 ± 0.82 ^μA^, *P* = *0*.*001*) however, this value was significantly lower than the ICTs at this stage (120 DPI), (79.16 ± 4.13 ^μA^*vs*. 42.82 ± 3.61 ^μA^, *P* = *0*.*001*) (Figure 4A).

There were no significant differences between the tissue resistance to direct electrical current (TRDEC) of the ITTs and ICTs just before the operation (185.25 ± 4.74 ^micro-ohm (μΩ)^*vs*. 184.05 ± 6.88 ^μΩ^, *P* = *0*.*927*) however, the TRDEC significantly decreased in both groups at 7 DPI. At this stage the TRDEC of the ITTs was significantly higher than the ICTs (15.05 ± 1.39 ^μΩ^*vs*. 4.45 ± 0.99 ^μΩ^, *P* = *0*.*001*). This decrease in the TRDEC continued to 14 DPI and the differences were statistically significant between the ITTs and the ICTs (10.85 ± 2.94 ^μΩ^*vs*. 2.75 ± 1.37 ^μΩ^, *P* = *0*.*001*). From 21 DPI to the end of the experiment, the TRDEC of both groups significantly increased at weekly intervals but at all intervals the measured value of the ITTs was significantly higher than the ICTs (*P* = *0*.*001* for all comparisons at weekly intervals) so that at 120 DPI the TRDEC of the ITTs was still significantly higher than the ICTs (72.55 ± 6.19 ^μΩ^*vs*. 43.15 ± 5.03 ^μΩ^, *P* = *0*.*001*). However, at this stage the TRDEC of the ITTs was significantly lower than those in the 0 DPI (*P* = *0*.*001*) (Figure 4B).

#### Dry matter content, water uptake and water delivery

There were significant differences between the dry matter content of the ITTs and the ICTs (30.38 ± 2.22% *vs*. 17.75 ± 2.05%, *P* = *0*.*001*) at 120 DPI. The ITTs showed a higher rate of water uptake compared to the ICTs so that the index of water uptake of the ITTs was significantly lower than the ICTs at 120 DPI (0.126 ± 0.011 *vs*. 0.591 ± 0.19, *P* = *0*.*001*). However, at this stage the ITTs showed significantly higher index of water uptake than their normal contralateral tendons (0.126 ± 0.011 *vs*. 0.050 ± 0.006, *P* = *0*.*001*) (Figure 4E).

At this stage, lower rate of the water delivery was seen in the ITTs compared to the ICTs so that the ITTs had significantly a lower index of water delivery compared to the ICTs (0.085 ± 0.009 *vs*. 0.293 ± 0.042, *P* = *0*.*001*). However, the index of water delivery of the ITTs was still significantly higher than their normal contralateral tendons at 120 DPI (0.085 ± 0.009 *vs*. 0.026 ± 0.007, *P* = *0*.*001*) (Figure 4F).

#### Gross pathological findings

**Figure 5:**
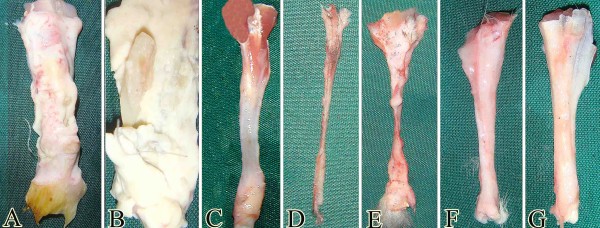
**Gross pathological findings (Results section: Gross pathological findings).** A severe inflammation was seen after 10 days following surgical implantation of the collagen implant (**A**). This healing tendon was cut longitudinally to find the remnants of the collagen implant in the injured area at 10 DPI. The granulation tissue was formed around this implant and the size of the implant was decreased (**B**). After 30 days following injury, the collagen implant was completely absorbed at gross level and a transparent but tendinous like tissue was formed in the injured area (**C**). At 120 DPI, the ICTs showed muscle fibrosis and disuse atrophy. The tendon edges were not connected by the newly regenerated tendinous tissue and the transverse diameter of the injured area in this group was decreased (**D&E**). The gross appearance of the tendons is hyperemic and a loose areolar connective tissue has filled the gap. Generally this tissue is not similar to the tendon and is more similar to an unorganized fascia (**D&E**). At this stage, the ITTs was completely formed and no remnant of the collagen implant was seen in the defect. Also the gross appearance of the newly regenerated tendon in the defect (**F**) is similar to the NCTs (**G**). No characteristic muscle fibrosis or atrophy in the ITTs is seen (**F**).

At 10 DPI, the transverse diameter of the ITTs characteristically increased to more than 3 folds compared to their normal contralateral tendons (Figure 5A). After the longitudinal incision on the ITTs, more than 75% of the initial size of the collagen implant still persisted (Figure 5B). The inflammation was still prominent and granulation tissue covered the defect and the implant (Figure 5B). At 30 DPI the collagen implant was completely absorbed and no remnant of this bio-implant was seen at the macroscopic level (Figure 5C). The newly regenerated, transparent connective tissue filled the gap. No tissue reaction or signs of inflammation were seen in these tendons at this stage (Figure 5C).

At 120 DPI, The ICTs showed severe hyperemia and the peri-tendinous adhesion developed and the gap was still present at this stage (Figure 5E). However a loose areolar connective tissue filled the gap (Figure 5D,E). The newly regenerated tissue was not morphologically comparable to tendon (Figure 5D *vs*. Figure 5G). It was transparent and the margins between the newly regenerated tissue in the defect area, and the peri-tendinous fascia, were not distinguishable (Figure 5D). In these animals, peri-tendinous adhesion was expanded to the gastrocnemius muscle so that the muscle fibrosis was diagnostic in these animals (Figure 5E). Generally, the transverse diameter of the ICTs was lower than their normal contralateral and the gastrocnemius muscle was atrophied so that the size and shape of the muscle were altered (Figure 5D). Unlike the control lesions, the ITTs, showed a better gross appearance (Figure 5F). The defect area was completely filled with the dense newly regenerated tendinous tissue with the characteristic margins. The peri-tendinous adhesion although developed but it was not characteristic and the tendon could move in its space between the gastrocnemius and calcaneal tuberosity. No muscle atrophy or fibrosis was seen in the treated animals. The transverse diameter of the ITTs was lower but comparable to their normal contralateral tendons and this difference was not characteristic at the gross level (Figure 5F *vs*. Figure 5G).

#### Histopathological findings

**Figure 6:**
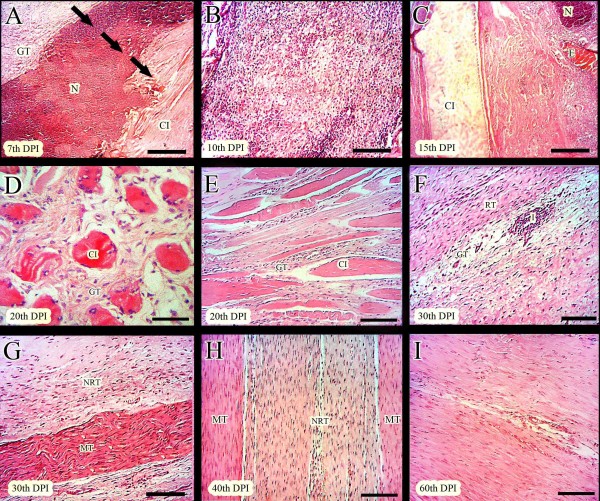
**Histopathological findings (Part 1), mechanism of host-graft interaction (Results section: Histopathological findings).** At 7 DPI, the inflammatory cells infiltrated inside the implant (**A**). Three parts are seen. The direction of the healing response is shown (arrows). Granulation tissue is formed around the implant. The inflammatory cells are in the middle part i.e. in the necrotic area. The third part is the collagen implant and no cell is seen in this area (**A**). At 10 DPI, the inflammatory cells are distributed all over the implant (**B**). At 15 DPI, different responses to implant are seen (**C**). In the left side, the implant (CI) is seen with no inflammatory response around it. On the right side, edema (**E**), neutrophil accumulation (N) and chronic inflammation is seen (**C**). At 20 DPI (**D&E**), the remnants of the implant are present with the lied tenoblasts around them. The granulation tissue is formed around these remnants. Inflammation was subsided and the remnants acted as scaffolds and aligned the new tissue (**D&E**). At 30 DPI, two parts in the injured tendons could be seen (**F&G**). No remnant of the implant was seen and the newly regenerated immature tendon at 20 DPI (**E**) was matured at this stage (**F&G**). The remnants of the collagen implant that were seen at 20 DPI, were absorbed and substituted by the newly regenerated tissue at this stage (**F&G**). At 40 DPI, this description is more likely to be characteristic and the newly regenerated tissue at the center of the field is aligned along the direction of the previously regenerated more mature tendinous tissue at the corners (**H**). At 60 DPI, all of the collagen fibers are mature and a homogenous tendon is formed (**I**). (**H&E**, Scale Bar: **A,C** =125 μm, **B** = 90 μm, **D** = 25 μm, **E-I** =50 μm).

In the pilot samples of the treated animals, seven days after injury, the collagen implant was invaded by the neutrophils and macrophages and they were invading the peripheral surfaces of the collagen implant and degrading it. However, some parts of the collagen implant were degraded by the inflammatory cells, at this stage, but they were not able to degrade all parts of the implant and most parts of the collagen implant were still present. These inflammatory cells were infiltrated in the newly regenerated granulation tissue surrounding the collagen implant and the inner parts of the collagen implant (Figure 6A). Three days later, the density of the inflammatory cells of the implant was reduced but they were generally distributed all over the collagen implant. Neutrophils, macrophages, lymphocytes, plasma cells and migrating fibroblasts were present in the injured area with the neutrophils as the dominant inflammatory cells (Figure 6B). At 15 DPI, the inflammation was subsided to few small foci and the granulation tissue started to mature. However, some parts of the collagen implant were surrounded by granulation tissue but no inflammatory cells were present around them. Edema and inflammation were still present at this time and size of the remnants of the degrading collagen implant was still large. The granulation tissue was almost aligned in the direction of these remnants of the collagen implant. Fibroblasts were the predominant cells at this stage however they were mostly immature (Figure 6C). At 20 DPI, the remnants of the degrading collagen implant laid in an aligned manner in only one direction, just between the line of the stress between the gastrocnemius muscle and calcaneal tuberosity of the tars and they acted as the intrinsic scaffold for the newly regenerated tissue. Few fibroblasts surrounded these remnants but they were not able to invade or proliferate through them. No signs of rejection were seen at this stage because the granulation tissue developed between them, the fibroblasts were aligned along the remnants of the collagen implant and vessels were present in the injured area. In addition inflammatory cells did not infiltrate around the newly regenerated vessels or the remnants of the collagen implant at the injured area. At this stage the histologic appearance of the injured area was similar to fibroplasia phase of tendon healing except the remnants of the collagen implant were still present in the tissue section (Figure 6D&E).

**Figure 7:**
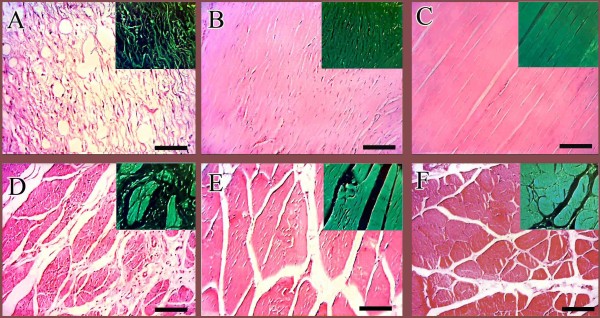
**Histopathological findings (Part 2) 120 days after injury (Results section: Histopathological findings).** There is fatty degeneration in the injured area of the ICTs. The density of the collagen fibers is extremely low and they are not aligned in a unidirectional pattern. These fibers with their cells laid along them are immature and generally the characteristics of this tissue are similar to the fascia than the tendon (**A**). On the other hand, in the ITTs, the collagen implant was completely absorbed at 120 DPI. The collagen fibers have high density with an aligned direction in the line of stress between the muscle and calcaneus. No obvious degeneration is seen and the cellular and collagenic structures are highly matured (**B**) similar to the intact tendons (**C**). The gastrocnemius muscle in the injured control legs shows muscle atrophy with the newly regenerated granulation tissue around them, suggesting both the atrophy and fibrosis (**D**). No muscle atrophy or fibrosis are seen in the treated lesions (**E**) and the pattern of the muscle fibers is almost similar to the muscle of the intact legs (**F**) than the control ones (**D**) (**H&E**, Scale Bar =50 μm).

At 30–40 DPI, the newly regenerated immature granulation tissue that was seen around the remnant of the collagen implant at 20 DPI, became mature and the remnants of collagen implant were absorbed and substituted by the newly regenerated aligned collagen fibers. No characteristic inflammation was evident in the injured area at these times (Figure 6F-H). At 60 DPI the immature and mature tendinous tissue that was observed at 40 DPI, were completely homogenized and matured so that all parts of the injured area was filled with the aligned and matured regenerated tendon (Figure 6I). At 120 DPI, the collagen fibers were denser and compact having fewer fibroblasts that were mostly matured. The small blood vessels were not evident in the injured area and all parts of the regenerated tissue showed the characteristics of the tendon (Figure 7B). At this stage the control tendons showed fatty degeneration, muscle fibrosis, peri-tendinous adhesions and the newly regenerated tissue had characteristics similar to loose areolar connective tissue (Figure 7A,D). No tendinous nature was evident in the injured area of the control lesions. Although the ITTs had similar characteristics to normal tendons, however, their cellularity was higher, the collagen fibers were not as dense as the normal tendons and no characteristic crimp pattern was seen in these tendons (Figure 7C).

## Discussion

The results of the present study showed that the tridimensional collagen implant was cytocompatible, biodegradable and biocompatible and was effective in improving the healing quality after acute tendon injury in an experimentally induced large tendon defect model in rabbits. The major merit of this study was that, this implant has been tested *in vivo* so that with a well-designed pilot and experimental study we were able to explain the mechanism of action of this implant on tendon healing. In addition, we followed the immune activity of the body in response to the implanted collagen prosthesis from the early stages to four months after injury by histopathological and hematological studies.

Simply, the ability of this highly aligned tridimensional collagen implant to improve tendon healing was due to the modulation effects of this bio-implant in activating the inflammatory and fibroblastic cells, attracted them into the defect area and controlled them in order to debride and proliferate throughout the collagen implant in the defect area.

Compared to the ICTs, the presence of higher transverse diameter and temperature in the injured area together with the higher infiltration of the inflammatory cells in the injured area of the treated lesions during the first 14 DPI, suggest that a higher inflammatory reaction has commenced, the healing response has been motivated by the collagen implant and the metabolism of the injured area has increased. At earlier stages of the healing, the ITTs had a more obvious inflammatory reaction compared to the ICTs but at 120 DPI, the gross morphologic, histopathologic, and biochemical characteristics of the ITTs were significantly more approximate to the intact tendons. It seems there is a strong correlation between the inflammatory response and tissue remodeling in tendon healing and also it revealed that; although the severe inflammatory reaction has been developed in response to the collagen implant, but this immune response was due to the remodeling effect of the collagen implant, not its rejection. It has been postulated that inflammation has a major role in tendon healing and if immune response does not properly develop after the injury, the healing response would be poor and no effective healing could be expected [5,24].

One of the major limitations of the tendon healing is a development of the peri-tendinous adhesion because it harms tendon to have its normal function [25,27-29]. Compared to the ICTs, less peri-tendinous adhesion and lower muscle fibrosis increased the ability of the ITTs to move in their subcutaneous space and these resulted in better weight bearing and physical activity with lower pain scoring on the injured limbs of the treated animals. Reduction in the peri-tendinous adhesion in the injured area had a strong correlation with the improved physical activity and weight bearing capacity and these factors, had a major role in aligning the regenerated tendinous tissue [25,28,29]. Possibly the healing ability of the ITTs was concentrated in the defect area and improved the morphological characteristics of the regenerated tissue. Unlike the ITTs, in the ICTs, the healing ability was randomly developed and the peri-tendinous fibroblasts proliferated not only in the injured area, but also they randomly invaded into the peri-tendinous tissues such as skin, subcutaneous fascia and muscle and proliferated and manufactured a haphazard granulation tissue throughout these structures. Thus, the potential of the healing response of the ICTs was divided into different regions so that the loose areolar connective tissue that was far from the tendinous structure, had formed in the defect area. By developing the muscle fibrosis and peri-tendinous adhesion, in the control lesions, the physical activity of the control animals was reduced and this resulted in developing muscle atrophy in these animals. It has been shown that the physical activity and weight bearing on the injured limb, has a significant correlation with collagen production and density of the injured area of the tendon [10,12,24].

Lower DTEC and higher TRDEC together with a higher transverse diameter of the injured area of the ITTs, specifically during the initial weeks after injury, could comprehensively explain that; unlike the ICTs, these significant changes were due to the higher tissue density of the ITTs in the defect area. On the other hand, inflammation and edema result in increased DTEC and reduction in TRDEC. To prove these statements, we have tested some biophysical characteristics as indices of collagen content of the regenerated tissues at 120 DPI. Compared to the ICTs, the ITTs had a higher dry matter content. They also showed almost similar patterns of water delivery and water uptake characteristics with their normal contralateral tendons.

This bio-implant was not rejected at acute or chronic stages. The histopathological findings demonstrated that the absorption mechanism of this implant was not correlated with the immune activity in response to implantation of the autografts and xenografts described by Veillette et al. [39]. They have stated that the response to antigenic tissue is predominately characterized by fibroblast and mononuclear cell infiltration with only modest inflammation and only partial matrix replacement of the graft. To describe the efficiency of the immune system in response to xenografts, they implanted the xenogenic based porcine patellar tendon, harvested from pigs to three different transplantation modality in rabbits including subcutaneous, medial collateral ligament mid-substance gap and entire medial collateral ligament complex.

Their results showed that after 6 weeks of injury, a vigorous inflammatory response was observed towards xenogenic PPT. Increased cellularity with infiltration of extensive neutrophils, lymphocytes, plasma cells, macrophages and foreign body giant cells indicated that the PPT was highly antigenic. They also reported that by three weeks after transplantation, in the pilot animal models, the xenografts tissue was rapidly absorbed and replaced by an amorphous scar tissue. They concluded that it is not yet practical to use xenografts as scaffolds for replacement of tendons or ligaments [39]. Our results unlike Veillette et al. [39] demonstrated that the pattern of absorption of the collagen bio-implant in the present study was more close to the autografts than xenografts. However, our implant was xenogeneic based obtained from the bovine tendon. The implant activated the inflammatory reaction and significantly improved the immune response compared to the control animals. However, the implant was not completely absorbed after 3 weeks and some parts of it were present in the injured area. At this stage, the remnants of the collagen implant had a major role in aligning the newly regenerated tissue. No foreign body or Langhans giant cells were present in the acute or chronic inflammatory phases of the present study. Moreover, similar to Veillette et al. [39] observations, the highest rate of inflammation existed in the first two weeks post-injury but no inflammatory reaction in the injured area of the treated animals was observed at six weeks after injury induction.

Such differences could be due to the methods of preparation of the collagen implant. Veillette et al. [39] used the xenograft but we extracted the collagen molecules from the xenograft, purified the collagen type I, reformed it into newly aligned collagen fibers and constructed a new architecture of the collagen based bioimplant. Therefore, the collagen purity of our scaffold was highly specific for type I collagen and no other tissue elements were present in our implant. Although collagen has been shown to increase the immune reaction but this chemo-attracting effect has been shown to be the remodeling reaction, not the rejection [40]. This statement is in accordance to Alman et al. [40] who implanted both the cellular and acellular porcine small intestinal sub-mucosa (SIS) in the subcutaneous area of mice. They indicated that xenogeneically implanted mice showed an acute inflammatory response followed by chronic inflammation and ultimately graft necrosis, consistent with rejection. Acellular SIS implanted mice, however, showed an acute inflammatory response that diminished so that the graft ultimately became indistinguishable from the native tissue. These observations are consistent with graft acceptance. They concluded that acellular SIS elicits an immune response that is consistent with a remodeling reaction rather than rejection.

Although the immune response was initiated by the collagen implant, however, the implant was completely substituted by the newly regenerated tissue that was morphologically and biochemically comparable to intact tendons. No signs of tissue rejection were seen and the collagen implant was well tolerated by the animals so that their hematological parameters were normal at 120 days after injury. All animals were clinically normal and no unexpected death was seen in the treated animals. In addition, application of the collagen implant in the injured area did not have deleterious effects on weight bearing capacity and did not result to unusual pain. Therefore, the outcome of the treated animals was acceptable and it is logic to test these types of tissue engineered implants in the future investigations.

Such investigation could possibly be a start point in the application of the tissue engineered collagen based bio-implants *in vivo* and it is a fact that some limitations affect the impact of these types of studies. Interestingly, it is imperative for future studies, to investigate the role of collagen based tissue engineered implants on the molecular aspects of tendon healing. With regards to the biophysical characteristics of this bioimplant such as water uptake and water delivery capacities, it would be interesting to assemble those agents that exert beneficial effects on the healing potential of connective tissues such as growth factors, glycosaminoglycans and tenoblastic stem cells with this bioimplant and investigate their effectiveness on extensive tendon injuries *in vivo*.

## Conclusion

The tridimensional hybridized xenogeneic based collagen implant was able to improve the healing quality of the injured tissue by its effectiveness on the inflammation, proliferation and remodeling phases of tendon healing in an experimentally induced tendon defect model in rabbits. This bio-implant was cytocompatible, biocompatible, and biodegradable. It facilitated the continuity of the injured tendons, decreased the peri-tendinous adhesion, and improved the muscle activity and clinical condition of the injured animals. No signs of rejection were seen in the injured area. This experiment with its limitations could possibly be considered as the initial stride in the field of application of the tissue engineered xenograft based implants on tendon transplantations. Future investigations could elucidate whether this bio-implant is able to be applied in clinical practice or not.

## Abbreviations

ITT: Injured treated tendon; ICT: Injured control tendon; NCT: Normal contralateral tendon; DTEC: Direct transmission electrical current; TRDEC: Tissue resistance to direct electrical current; DPI: Days post injury.

## Competing interests

The authors have declared that no competing interests exist.

## Authors’ contributions

AO and AM wrote this manuscript. All authors participated in this research, read and approved the final manuscript.
